# Primary Angioplasty for Cardiac Allograft Vasculopathy Presenting as ST-Elevation Acute Myocardial Infarction during Endomyocardial Biopsy

**DOI:** 10.1155/2013/606481

**Published:** 2013-08-27

**Authors:** Bruno Ramos Nascimento, Thalles Oliveira Gomes, Júlio César Borges, Guilherme Rafael Sant'Anna Athayde, Sílvio Amadeu de Andrade, Maria da Consolação Vieira Moreira

**Affiliations:** ^1^Serviço de Hemodinâmica do Hospital das Clínicas da Universidade Federal de Minas Gerais, 30130-100 Belo Horizonte, MG, Brazil; ^2^Serviço de Hemodinâmica do Hospital Universitário São José-INCOR Minas, 30140-073 Belo Horizonte, MG, Brazil; ^3^Serviço de Transplante Cardíaco do Hospital das Clínicas da Universidade Federal de Minas Gerais, 30130-100 Belo Horizonte, MG, Brazil

## Abstract

Cardiac allograft vasculopathy is still a major issue, with significative mortality in heart transplant patients, and the best therapeutic options are not yet established. The progressively higher survival rates after transplantation have made it a major concern. This is a case report about a patient who underwent cardiac transplantation due to chagasic cardiomiopathy. During an endomyocardial biopsy more than 2 years after the transplant, the patient arrested in ventricular fibrillation, with ST-elevation in anterior leads after defibrillation. The angiography showed total occlusion of proximal left anterior descending artery, promptly treated with primary angioplasty, with excellent angiographic and clinical results.

## 1. Introduction

The cardiac allograft vasculopathy (CAV) is a major cause of morbidity and mortality in patients undergoing cardiac transplantation, and, as the survival rates increase, there has been a growing concern about its clinical management. Sudden death and graft ventricular dysfunction are some of the main manifestations, with cases presenting as acute coronary syndromes reported in the literature. This is a report of a patient after cardiac transplant due to Chagas disease that developed cardiac arrest immediately after endomyocardial biopsy, with postdefibrillation electrocardiogram suggestive of ST-elevation acute myocardial infarction in the anterior wall, successfully treated with primary angioplasty.

## 2. Case Report

Patient, DMS, male, 40 years, had undergone heart transplantation 2 years and 3 months ago due to Chagas cardiomyopathy. The donor was a young male, without any known cardiovascular risk factors. The patient remained stable until a month ago, when he was admitted to the hospital with moderate dyspnea. Late allograft rejection was diagnosed, histopathologically graded as 3A by endomyocardial biopsy. The patient was subjected to intravenous pulse therapy with corticosteroids and was discharged asymptomatic, on tacrolimus 6 mg/d, mycophenolate sodium 1440 mg/d, prednisone 40 mg/d, simvastatin 40 mg/d, and benzimidazole 300 mg/d. Echocardiogram performed during hospitalization showed normal left ventricular (LV) function, with left ventricle ejection fraction (LVEF) = 80%.

**Figure 1:**
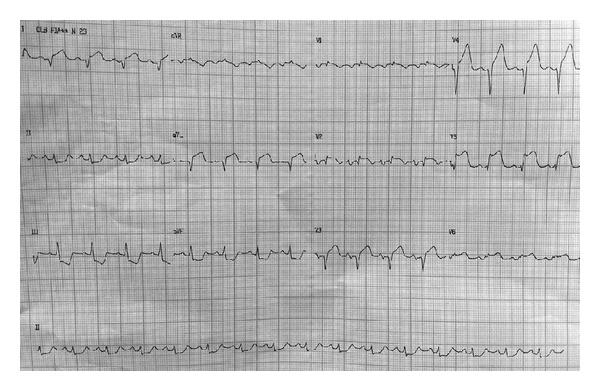
Postarrest electrocardiogram showing ST-segment elevation of up to 5 mm in V1 to V6, DI, and aVL, with ST downslope up to 2 mm in inferior leads.

After discharge the patient was referred for elective endomyocardial biopsy. During the procedure, immediately after removal of the third fragment without complications, he arrested due to ventricular fibrillation and was immediately defibrillated. The electrocardiogram (EKG) after the arrest showed ST-segment elevation up to 5 mm in V1 to V6, DI, and aVL, with ST downslope up to 2 mm in inferior leads (Figure 1). There was no chest pain complaint. Emergency echocardiogram was then performed in the CathLab, and showed LVEF = 40%, normal LV diameters, interventricular septum akinesis, severe hypokinesis of the mid-apical segments of the anterior-septal and lateral walls, and severe hypokinesis of the apical segment of the inferior wall, apex, and anterior wall.

**Figure 2:**
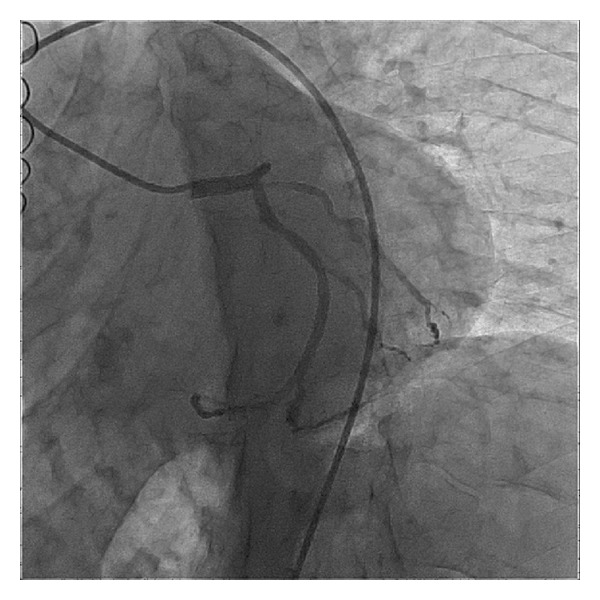
Angiography showing total occlusion of the proximal left anterior descending artery, with moderate lesions in the mid-circumflex artery and in the proximal first marginal branch.

**Figure 3:**
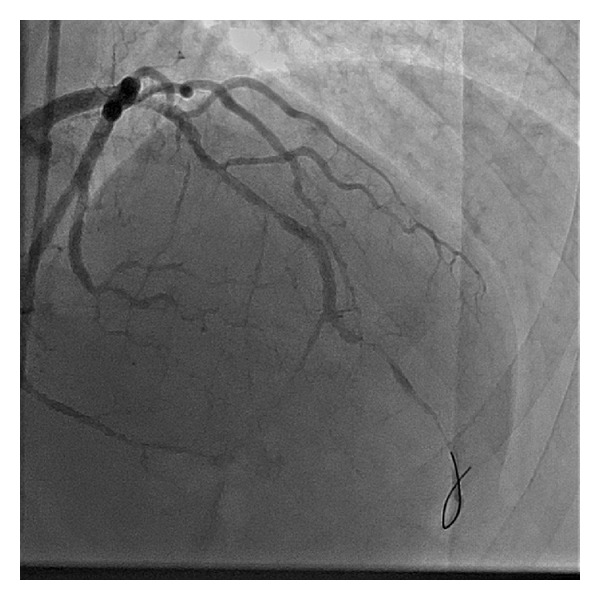
After left anterior descending artery (LAD) recanalization: severe diffuse disease in the proximal and mid LAD, and severe spasm in its distal third.

**Figure 4:**
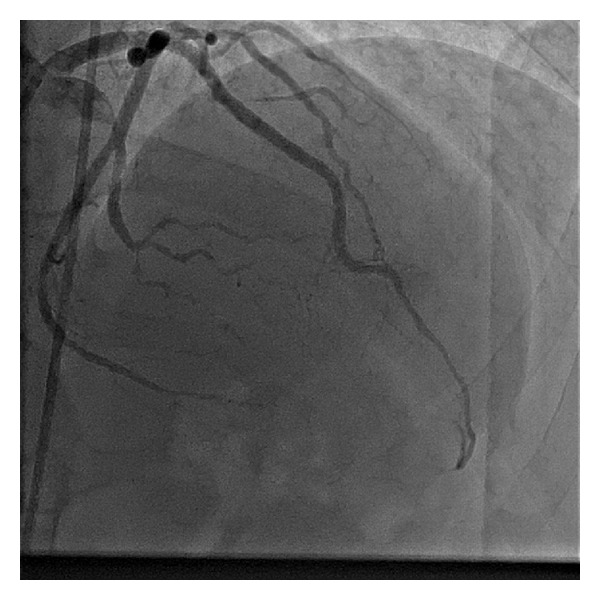
Final result of the primary angioplasty, showing angiographic success.

Emergency coronary angiography was performed and showed total occlusion of the proximal left anterior descending artery (LAD), with moderate lesions (40%) in the mid-circumflex artery and in the proximal first marginal branch (Figure 2), and total occlusion of the hypoplastic proximal right coronary artery. The patient underwent primary angioplasty for the LAD: a 0.014 floppy guidewire was passed through the occlusion, followed by predilatation with a 2.5  ×  15 mm balloon. After the vessel recanalization, severe diffuse disease was observed in the proximal and mid LAD, with severe spasm in its distal third (Figure 3). Implantation of three overlapped bare metal stents (BMS): 3.0  ×  15 mm, 3.0  ×  30 mm, and 3.0 × 9 mm was successfully performed. The spasm was reversed after administration of intracoronary nitroglycerin, and a satisfactory angiographic result was achieved (Figure 4, Supplementary Video 1 (see Supplementary Material available online at http://dx.doi.org/10.1155/2013/606481)).

After the procedure, there was regression of more than 50% of the ST segment upslope. The patient was in Killip class 2 and improved after anticongestive therapy, in the first 24 hours. No significant electrolyte abnormalities were observed. He remained stable during hospitalization and was discharged from the intensive care unit four days after the event. The biopsy showed rejection grade 2B, and systemic corticosteroid pulse therapy was then initiated. New echocardiogram performed before discharge showed significant improvement of the LV function: LVEF = 52%, mild hypokinesis of the septal and anterior walls, and akinesia of the apex. After discharge, the patient remained asymptomatic and was maintained on high doses of oral corticosteroids, for subsequent gradual withdrawal. Mycophenolate sodium was replaced by everolimus in the immunosuppressive scheme.

## 3. Discussion

This is the report of an unprecedented case in the literature of acute myocardial infarction during endomyocardial biopsy, treated with primary angioplasty. Endomyocardial biopsy is the method of choice for graft rejection assessment after heart transplantation, with reported complication rates of less than 1% [1], mostly arrhythmic and mechanical events, with a previously published report of procedure-related myocardial infarction secondary to hematoma, caused by coronary fistula [2].

CAV is characterized by diffuse intimal thickening and luminal obstruction of the coronary arteries of the transplanted heart, immunologically mediated, and is the main cause of late graft loss and mortality after heart transplantation. The clinical manifestations vary widely, from graft dysfunction to sudden death, that may be related to acute events [3, 4]. It occurs in approximately 30% of patients by 5 years and 50% by 10 years, and early detection is important, allowing modifications to medical therapy before the progression to the stage in which revascularization is required. Routine screening for CAV in transplant recipients, traditionally by invasive coronary angiography (ICA), has been recently recommended, due to the limitations of the available noninvasive tests. Recent developments in intravascular imaging techniques, specifically intravascular ultrasound and optical coherence tomography, may now allow the validation of new criteria for early detection of subangiographic CAV [4]. In our institution there is no recommendation for routine ICA in the first 2 years after cardiac transplantation in the absence of clinical or echocardiographic manifestations of CAV. 

In advanced stages, angioplasty has suboptimal outcomes, with higher rates of restenosis compared to interventions in native vessels, and some series have demonstrated the superiority of drug-eluted stents over BMS also in these cases, with lower rates of restenosis and target vessel revascularization [5]. In this case, the procedure was performed with BMS due to the Brazilian Public Health Insurance System (SUS), that does not cover either drug-eluted stents or intravascular imaging modalities for guiding stent implantation, such as intravascular ultrasound or optical coherence tomography. Moreover, there are still some doubts regarding the safety of drug-eluting stents in the ST-elevation myocardial infarction, given the higher rates of stent thrombosis in some series, possibly related to late malapposition [6, 7].

In this case, some hypotheses were raised for the cause-and-effect relation between CAV and acute myocardial infarction. The patient probably had previously developed CAV, and acute vessel occlusion occurred during the biopsy due to physical and metabolic stress, leading to ventricular fibrillation. The denervation of the transplanted heart makes this diagnosis even more challenging due to the lack of chest pain, as the echocardiogram performed in the cathLab was a crucial tool for decision making. Other plausible possibility is that ventricular fibrillation was induced by the bioptome, and thrombus formation on the diseased vessel segment was secondary to hemodynamic deterioration induced by the ventricular arrhythmia. The percutaneous approach with BMS was performed successfully, with excellent angiographic and clinical outcomes in this unprecedented situation, and the patient remains clinically stable in the late followup.

## 4. Conclusion

In this unprecedented case report, in which allograft vasculopathy presented as acute occlusion of the LAD during myocardial biopsy—a complication not previously reported in the literature—the patient was successfully treated with primary angioplasty, with angiographic success and clinical improvement. 

## Supplementary Material

Primary angioplasty procedure, as described in the case report. After pre-dilatation of the occluded left anterior descending wall (LAD), we observed severe disease in its proximal and mid segments, and spasm in its distal third. Three bare-metal stents were deployed in the proximal and mid LAD, with satisfactory angiographic result. After administration of intracoronary nitrate, the spasm was almost completely reversed.
